# Systematic use of protein free energy changes for classifying variants of uncertain significance: the case of IFT140 in Mainzer-Saldino Syndrome

**DOI:** 10.3389/fmolb.2025.1561380

**Published:** 2025-04-23

**Authors:** Macarena Gajardo, José Luis Guerrero, Bárbara Poblete, Esperanza Bayyad, Ignacio Castro, Jorge Maturana, Jaime Tobar, Víctor Faúndes, Paola Krall

**Affiliations:** ^1^ Facultad de Medicina, Universidad de Chile, Santiago, Chile; ^2^ Servicio de Nefrología, Hospital Luis Calvo Mackenna, Santiago, Chile; ^3^ Escuela de Tecnología Médica, Facultad de Medicina, Universidad Austral de Chile, Valdivia, Chile; ^4^ Instituto de Informática, Facultad de Ciencias e Ingeniería, Universidad Austral de Chile, Valdivia, Chile; ^5^ Servicio de Pediatría, Hospital de Arica, Arica, Chile; ^6^ Laboratorio de Genética y Enfermedades Metabólicas, Instituto de Nutrición y Tecnología de los Alimentos, Universidad de Chile, Santiago, Chile; ^7^ Laboratorio de Nefrología, Facultad de Medicina, Universidad Austral de Chile, Valdivia, Chile; ^8^ Centro de Investigación Clínica Avanzada (CICA)-Hospital Luis Calvo Mackenna, Santiago, Chile

**Keywords:** missense variant, bioinformatics, free energy changes, Mainzer-Saldino Syndrome, ciliopathy, IFT140

## Abstract

**Introduction:**

Advanced genetic strategies have transformed our understanding of the genetic basis and diagnosis of many phenotypes, including rare diseases. However, missense variants (MVs) are frequently identified and often classified as variants of uncertain significance (VUS). Although changes in protein free energy (ΔΔG) were recently proposed as a tool for VUS classification, no objective cut-offs exist to distinguish between benign and pathogenic variants.

**Methods:**

We utilized the computational tool mCSM to calculate ΔΔG and predict the impact of MVs on protein stability. Specifically, we systematically analyzed the ΔΔG of MVs in IFT140 to identify those potentially pathogenic and associated with Mainzer-Saldino syndrome (MSS). To this end, we evaluated ΔΔG in IFT140 MVs sourced from ClinVar, gnomAD, and MSS patients, aiming to resolve the diagnosis of MSS in a child with a novel homozygous IFT140 variant, initially reported as a VUS.

**Results:**

IFT140 MVs from MSS patients showed lower ΔΔG values than those reported in gnomAD individuals (−1.389 vs. −0.681 kcal/mol; p = 0.0031). A ROC curve demonstrated strong discriminative ability (AUC = 0.8488; p = 0.0002), and a ΔΔG cut-off of −1.3 kcal/mol achieving 50% sensibility and 90% specificity. The analysis of ClinVar IFT140 variants classified as VUS, showed that 75/323 (23%) presented ΔΔG values below the cut-off. In the child clinically suspicious of MSS, this cut-off allowed the reclassification of the VUS (IFT140:p.W80C; ΔΔG = −1.745 kcal/mol) as likely pathogenic, which confirmed the diagnosis molecularly.

**Conclusion:**

Our findings demonstrate that ΔΔG analysis can effectively distinguish potentially pathogenic variants in IFT140, enabling confirmation of MSS. The established cut-off of −1.3 kcal/mol showed strong discriminative power, aiding in the reclassification of VUS identified in IFT140. This approach highlights the utility of protein stability predictions in resolving diagnostic uncertainty in rare diseases.

## Introduction

Advanced genetic strategies have transformed our understanding of the basis and diagnosis of many diseases. Protein-truncating variants (PTV) are usually considered pathogenic due to their strong functional effects, with few exceptions. However, missense variants (MV) are more frequent than PTV and often classified as variants of uncertain significance (VUS) due to limited population frequency data and functional evidence, underscoring the need for improved methods of resolution (Richards et al., 2015), (Appelbaum et al., 2022).

Short-rib thoracic dysplasia 9 (SRTD9, OMIM#266920), or Mainzer-Saldino Syndrome (MSS), is an extremely rare ciliopathy affecting fewer than 1 in 1,000,000 individuals. MSS is characterized by chronic kidney disease (CKD), progressive vision impairment, with distinctive skeletal features such as cone-shaped epiphyses (Geoffroy et al., 2018). Around 20 MSS cases have been reported, involving homozygous or compound heterozygous variants in the Intraflagellar Transport 140 (*IFT140*) gene. IFT140 contains multiple WD40 repeat (WD) and tetratricopeptide-repeat (TPR) domains that enable the protein to interact with others to form complex structures, facilitating the transport of proteins along the cilia. Accurate interpretation of *IFT140* variants is essential for MSS diagnosis, guiding personalized treatment, avoiding unnecessary interventions, informing prognosis, and identifying at-risk family members (Walsh et al., 2024). The identification of novel MVs in *IFT140* has a high likelihood of being classified as VUS, delaying diagnosis and limiting their clinical utility (Chen et al., 2023).

Several methods have been developed to enable VUS reclassification. These include population allele frequencies, functional assays, and machine learning models, with structural models specifically providing insights into the underlying molecular mechanisms. Gibbs free energy (ΔG) is a thermodynamic measure used to quantify protein stability, based on the principle that structures tend to adopt more negative energy states. If the difference between the folded and native forms of a protein results in a more negative value, the process occurs spontaneously. In this context, the change in Gibbs free energy (ΔΔG) evaluates the difference between the Gibbs free energies of a wild-type and a mutant protein. A negative ΔΔG value indicates that the mutant protein is destabilizing (Sapozhnikov et al., 2023). Recently, the Association for Clinical Genomic Science (ACGS 2024) recommended the use of ΔΔG caused by MVs for their classification, based on a successful clinical experience (Caswell et al., 2022), (ACGS, 2024). However, the proposed ΔΔG cut-offs were subjectively determined as they did not consider the full spectrum of ΔΔG seen in both benign and pathogenic MVs (Caswell et al., 2022).

The mutation Cutoff Scanning Matrix (mCSM) was introduced in 2014 to predict MVs effects by assessing protein structure changes and stability using ΔΔG (Pires et al., 2014). It offers a computationally efficient, easy-to-use and well-validated approach with optimal correlation to experimental ΔΔG data. When analyzing amino acid substitutions in different human diseases, mCSM predicted a higher proportion of variants as destabilizing and demonstrated better predictive power compared to other structure-based tools (Choudhury et al., 2022). Herein, we aimed to systematically establish a ΔΔG cut-off for *IFT140* MVs using mCSM, which helped to confirm the molecular diagnosis of MSS in a child with a novel homozygous *IFT140* variant, initially classified as VUS.

## Materials and methods

First, we aimed to compare the ΔΔG seen in benign versus pathogenic *IFT140* MVs to establish a cut-off that allowed us to distinguish between these two groups and reclassify VUS deposited in ClinVar. For this purpose, we downloaded *IFT140* variants from ClinVar Miner (version 06-30–2024) with their ACMG classifications: P and LP were combined into the pathogenic (ClinVar-P) group, B and LB were combined into the benign (ClinVar-B) group, and VUS (ClinVar-VUS) were kept as a separate group (Henrie et al., 2018). Minor allele frequency (MAF) was assessed in each group using the gnomAD v4.1 database. Homozygous *IFT140* MVs were obtained from gnomAD, focusing on LB/B classifications according to ClinVar and assumed as controls (Chen et al., 2024). Homozygous or compound heterozygous *IFT140* MVs associated with MSS phenotype were collected and considered as cases (Geoffroy et al., 2018), (Patel et al., 2023), (Perrault et al., 2012), (Yeh et al., 2022), (Soyaltın et al., 2018), (Schmidts et al., 2013), (Oud et al., 2018). PTVs or synonymous *IFT140* variants were excluded. The PDB file for IFT140 (8BBG, 3.50 Å, chain B, positions 1–1462) was downloaded from UniProt and used to calculate ΔΔG for each MV using mCSM (https://biosig.lab.uq.edu.au/mcsm/) (Pires et al., 2014). Mean ΔΔG values from gnomAD and MSS variants were compared using Mann-Whitney U test, and Receiver Operating Characteristic (ROC) curve was constructed to define a ΔΔG cut-off. In addition, the distribution of variants in IFT140 was evaluated, focusing on the 7 WD and 9 TPR domains according to the information available on UniProt. We made a statistical analysis of the position within and outside these domains comparing gnomAD and MSS patients. Chi square test was considered to determine statistical significances of these distributions.

Second, we aimed to reclassify a novel homozygous *IFT140* MV seen in a patient with a phenotype compatible with MSS and considering the obtained cut-off, and to study the relevance of the affected residue for the syndrome. For these purposes, detailed clinical history and exome data were collected and informed consent was obtained from parents. The identified *IFT140* variant and all possible residue changes underwent systematic ΔΔG analysis using mCSM, to compare those values with the cut-off. The study was revised and approved by the Ethics Committee from the Servicio de Salud Los Ríos.

## Results

We obtained 908 genetic changes registered in ClinVar as variants compromising *IFT140*. These changes included 412 (45.4%) MVs, 230 (25.3%) synonymous variants, 181 (19.9%) intronic variants with different possible impacts, 49 (5.4%) rearrangements that involved *IFT140*, and only 36 (4.0%) PTVs.

**FIGURE 1:**
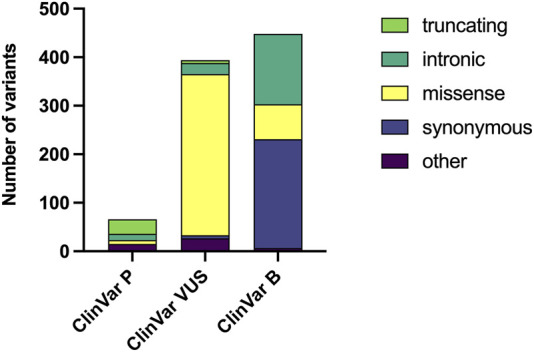
Distribution of genetic variants by pathogenicity classification and variant type. The number of genetic variants downloaded from ClinVar Miner are displayed, categorized by clinical significance as Pathogenic (P), Variant of Uncertain Significance (VUS), and Benign (B). Within each bar, the abundance of different variant types (e.g., truncating, missense) is represented.

When considering their classification, 66 variants were ClinVar-P, 394 variants were considered ClinVar-VUS, and 448 variants were ClinVar-B (Figure 1). In the ClinVar-P group, 43 (65.2%) variants were PTVs. MVs represented 84.3% of all variants in ClinVar-VUS, while they were found in 16.1% and 12.1% of the ClinVar-B and ClinVar-P groups, respectively.

The gnomAD database showed the MAF for 196 of all the MVs in the different ClinVar groups. The average MAFs in the ClinVar-P, ClinVar-VUS and ClinVar-B groups were 0.475, 0.250 and 38.76 per 10,000 individuals, respectively. Of note, 29 (49.1%) out of 59 MVs in the ClinVar-B group had a MAF of less than 1 in 10,000, while in the ClinVar-VUS group, this characteristic was observed in 130 out of 133 MVs.

**FIGURE 2:**
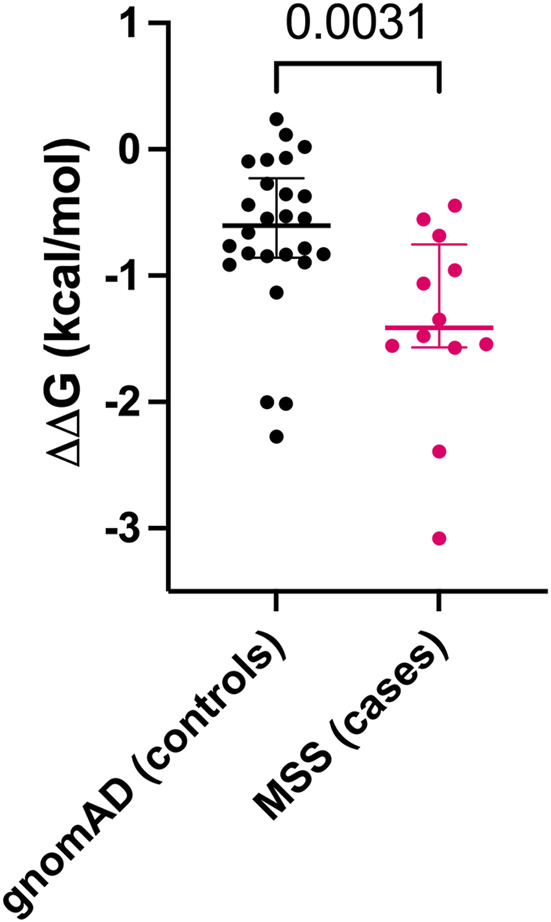
Comparison of ΔΔG values for missense variants (MVs) between gnomAD individuals (controls) and Mainzer-Saldino Syndrome (MSS) patients (cases). Each dot represents a single MV, with median values and interquartile ranges shown for both groups.

We calculated ΔΔG for gnomAD individuals with homozygous *IFT140* MVs (n = 26) and for MSS patients (3 homozygous and 9 heterozygous MVs). The mean ΔΔG for gnomAD MVs was −0.681 kcal/mol (95%CI: −0.937 to −0.426) and for MSS MVs it was −1.389 kcal/mol (95%CI: −1.872 to −0.907). The difference in ΔΔG values between gnomAD and MSS *IFT140* MVs was significant (U = 64, p = 0.0031) (Figure 2).

**FIGURE 3:**
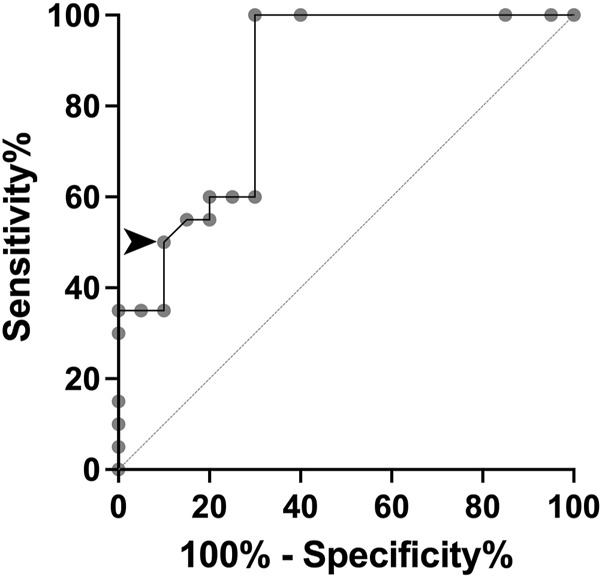
ROC curve based on ΔΔG values for missense variants (MVs) from gnomAD controls and MSS cases. The arrowhead indicates the established cut-off value to achieve 50% sensitivity and 90% specificity.

By using our ΔΔG data in gnomAD individuals and MSS patients, we constructed the ROC curve and obtained an AUC of 0.8488 (p = 0.0002), indicating an optimal discriminative ability to identify likely pathogenic MVs. The cut-off point was established at ΔΔG -1.3 kcal/mol, yielding a sensitivity of 50% and a specificity of 90% (Figure 3). For ClinVar groups, the average ΔΔG values were −1.432 kcal/mol (95%CI: −2.074 to −0.789), −0.798 kcal/mol (95%CI: −0.878 to −0.717), and −0.796 kcal/mol (95%CI: −0.953 to −0.640), for *IFT140* MVs classified as pathogenic, VUS, or benign, respectively. Of note, in the ClinVar-VUS group, 75/323 MVs had ΔΔG values below −1.3 kcal/mol (Supplementary Figure S1).

The analysis from gnomAD individuals and MSS patients regarding their MV positions within and outside WD and TPR domains showed that 7 out of 26 variants (27%) reported in gnomAD homozygous individuals and 5 out of 9 variants (56%) identified in MSS patients were in these domains. However, this difference did not reach statistical significance (p = 0.1255).

**FIGURE 4:**
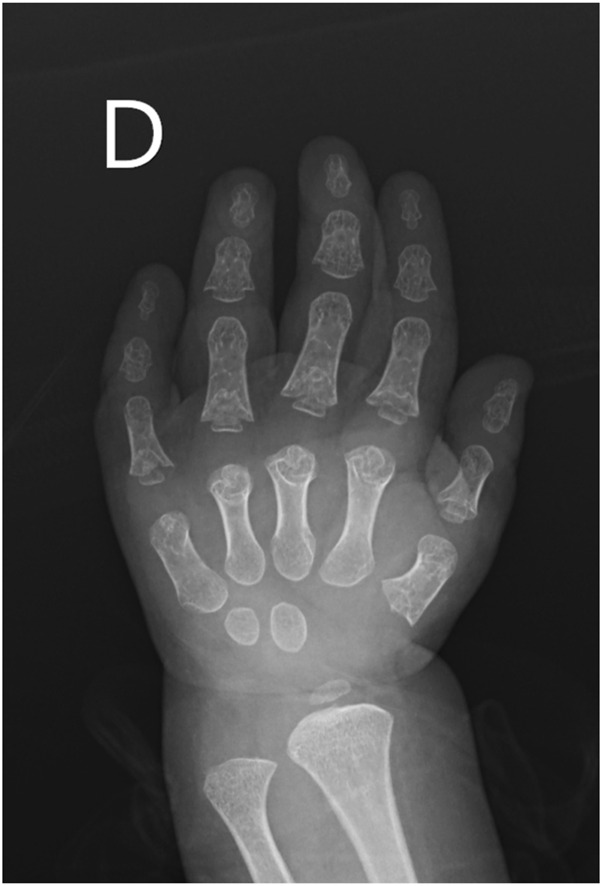
X-ray image of the right hand from the pediatric patient suspected of having Mainzer-Saldino Syndrome (MSS). The image reveals characteristic skeletal abnormalities associated with MSS (cone-shaped epiphyses).

A novel homozygous *IFT140* variant was discovered in a 1.5-year-old girl who was evaluated for CKD stage 4 with small kidneys without cystic pattern, hepatic fibrosis, neurological developmental delay, and unilateral moderate sensorineural hearing loss. The array comparative genomic hybridization was normal, *PKD1* analysis by long-range PCR sequencing was negative, but exome sequencing identified the *IFT140* c.240G>T (p.W80C) homozygous MV categorized as VUS. This variant was inherited from both heterozygous parents aged 34 and 43 years, who underwent abdominal ultrasounds, with no relevant findings observed, particularly in the kidney structures (Dordoni et al., 2024). Subsequent evaluations in the patient revealed retinal dystrophy, and phalangeal cone-shaped epiphyses in both hands (Figure 4), confirming the clinical diagnosis of MSS. The ΔΔG value for *IFT140*:p.W80C was −1.745 kcal/mol, and further systematic evaluation of the W80 residue indicated that all possible amino acid substitutions were destabilizing (Table 1).

**TABLE 1:** ΔΔG values obtained for all possible substitutions of the IFT140 W80 residue with the pdb file downloaded with the identifier 8BBG. The substitution W80C seen in the child has been highlighted in bold.

Index	Wild type residue	Position	Mutant residue	Predicted ΔΔG	Outcome
1	W	80	A	−3.989	Highly Destabilizing
2	W	80	V	−3.545	Highly Destabilizing
3	W	80	L	−3.277	Highly Destabilizing
4	W	80	G	−4.16	Highly Destabilizing
5	W	80	S	−3.146	Highly Destabilizing
6	W	80	T	−2.99	Highly Destabilizing
7	W	80	Q	−2.936	Highly Destabilizing
8	W	80	E	−3.537	Highly Destabilizing
9	W	80	C	−1.745	Destabilizing
10	W	80	R	−1.994	Destabilizing
11	W	80	P	−3.545	Highly Destabilizing
12	W	80	D	−3.632	Highly Destabilizing
13	W	80	F	−2.661	Highly Destabilizing
14	W	80	I	−3.277	Highly Destabilizing
15	W	80	H	−2.97	Highly Destabilizing
16	W	80	N	−2.932	Highly Destabilizing
17	W	80	M	−2.535	Highly Destabilizing
18	W	80	Y	−2.418	Highly Destabilizing
19	W	80	K	−2.516	Highly Destabilizing

## Discussion

Rare diseases are a heterogeneous group of conditions with an emerging global impact, a population prevalence of 3.5%–5.9% and with a genetic origin in almost 80% (Nguengang et al., 2020). The rate of VUS in genes associated with rare diseases is 41.5%, and MVs represent the largest proportion of them (86.6%) (Chen et al., 2023). Half of the *IFT140* benign MVs had a MAF<1/10,000 suggesting that rarity of a variant is not a reliable indicator of pathogenicity and reinforcing the need of additional methods for reanalysis.

In recent years, increasing interest in predicting the pathogenicity of MVs VUS using structure-based algorithms has emerged (Pan and Theesfeld, 2024). One such approach involves calculating the ΔΔG between the wild-type and variant residues; however, establishing a valid cut-off value is essential (Pires et al., 2014), (David and Sternberg, 2023). Recent studies have demonstrated that ΔΔG is a useful tool for evaluating other MVs in genes such as transcription factor FOXD2 and TBC1D31 which are now implicated in syndromic congenital anomalies of the kidney and urinary tract (Riedhammer et al., 2024; Saygılı et al., 2023), as well as NUP85, associated with steroid-resistant nephrotic syndrome (Şükür et al., 2025). In the field of protein stability, various computational tools aid in the assessment of MVs, including mCSM, Dynamut2 (Rodrigues et al., 2021), FoldX (Schymkowitz et al., 2005) and PremPS (Chen et al., 2020), among others. These tools evaluated stability, folding and dynamics of proteins. Additionally, molecular dynamics simulations offer valuable insights into atomic movements within a protein by modeling interatomic interactions (Hollingsworth and Dror, 2018).

The ΔΔG in *IFT140* MVs revealed significant differences between benign (gnomAD individuals) and pathogenic (MSS patients) variants. We established a cut-off value of −1.3 kcal/mol with an optimal sensitivity and specificity for *IFT140* MVs. When analyzing the totality of MVs in the ClinVar-VUS group, we identified that 23.2% had ΔΔG values below the cut-off value, suggesting that these might be re-classified as likely pathogenic.

The novel variant *IFT140*:p.W80C found in our female patient, initially classified as VUS, is a good example of the impact of our work. The child met all clinical features to be diagnosed with MSS (Soyaltın et al., 2018), and the ΔΔG value of −1.745 kcal/mol for p.W80C, below our calculated cut-off, confirms the deleteriousness of this variant. Additionally, the destabilizing values of all possible substitutions at this position indicate its intolerance to amino acid changes, which allowed us to reclassify the p.W80C as likely pathogenic, following ACGS 2024 recommendations (ACGS, 2024).

Our study has limitations in different aspects. Firstly, few patients with MSS and gnomAD *IFT140* homozygous individuals have been reported, which decreases the sensibility of our findings. However, this is the first study to our knowledge that systematically evaluates a ΔΔG cut-off value in patients with *IFT140* VUS, which has the potential to be used for the evaluation of other similar patients. Further calculations of ΔΔG cut-offs for other monogenic diseases are required to implement the recent ACGS 2024 recommendations of structural damage for MVs classification (ACGS, 2024).

Bioinformatic tools that evaluate ΔΔG have several limitations, many of which depend on structural data availability. Most require a high-resolution 3D protein structure, typically from Protein Data Bank (PDB). If the variant of interest is in a flexible or disordered region that is not well-captured in the structure, predictions can be unreliable. When no structure is available, these tools must rely on homology modeling, which introduces additional errors compared to crystal structures. Additionally, environmental effects are often poorly accounted for in these tools. Factors such as solvent interactions, post-translational modifications, *in vivo* physiological conditions, and protein-protein interactions are either oversimplified or completely ignored. This is particularly problematic when multiple pathogenic mechanisms coexist. Finally, ΔΔG prediction tools can yield conflicting results, as they rely on different algorithms and training datasets. To improve reliability, it might be reasonable to use multiple tools and compare their outputs.

By 2030, VUS in coding regions are expected to be resolved through several advancements, including refinements in variant classification standards, improved performance of computational variant effect predictors, the establishment of large-scale ΔΔG datasets, the development of hybrid *in silico* and experimental approaches, and enhanced data-sharing efforts that maximize the information gained from each newly sequenced individual and interpreted variant (Fowler and Rehm, 2024). Additionally, machine learning approaches will play a key role by integrating large-scale genomic, functional, and clinical data, improving predictive accuracy, and identifying complex patterns that traditional methods might overlook (Quazi, 2022; Khalifa and Albadawy, 2024).

In conclusion, the analysis of ΔΔG can aid in the evaluation of *IFT140* MVs for diagnostic purposes. The appropriate approach should include the assessment of clinical findings, the evaluation of genetic variants using meta-predictors or other assays, with ΔΔG serving as one of the components of the comprehensive strategy in patients suspected to have MSS or other rare diseases. Future advancements in variant classification standards, computational predictors, multiplexed assays, and machine learning approaches will further enhance the interpretation of ΔΔG and its role in variant assessment, especially those of uncertain significance.

## Data Availability

The datasets presented in this study can be found in online repositories. The names of the repository/repositories and accession number(s) can be found below: Figshare, doi: 10.6084/m9.figshare.28199999, https://figshare.com/articles/dataset/Kidney_Panel_Variants/28199999?file=51653081.

## References

[B1] ACGS (2024). ACGS best practice guidelines for variant classification in rare disease. Available online at: https://www.acgs.uk.com/media/12533/uk-practice-guidelines-for-variant-classification-v12-2024.pdf.

[B2] Appelbaum P. S. Burke W. Parens E. Zeevi D. A. Arbour L. Garrison N. A. (2022). Is there a way to reduce the inequity in variant interpretation on the basis of ancestry? Am. J. Hum. Genet. 109 (6), 981–988. 10.1016/j.ajhg.2022.04.012 35659933 PMC9247826

[B3] Caswell R. C. Gunning A. C. Owens M. M. Ellard S. Wright C. F. (2022). Assessing the clinical utility of protein structural analysis in genomic variant classification: experiences from a diagnostic laboratory. Genome Med. 14 (1), 77. 10.1186/s13073-022-01082-2 35869530 PMC9308257

[B4] Chen E. Facio F. M. Aradhya K. W. Rojahn S. Hatchell K. E. Aguilar S. (2023). Rates and classification of variants of uncertain significance in hereditary disease genetic testing. JAMA Netw. Open 6 (10), e2339571. 10.1001/jamanetworkopen.2023.39571 37878314 PMC10600581

[B5] Chen S. Francioli L. C. Goodrich J. K. Collins R. L. Kanai M. Wang Q. (2024). A genomic mutational constraint map using variation in 76,156 human genomes. Nature 625, 92–100. 10.1038/s41586-023-06045-0 38057664 PMC11629659

[B6] Chen Y. Lu H. Zhang N. Zhu Z. Wang S. Li M. (2020). PremPS: predicting the impact of missense mutations on protein stability. PLoS Comput. Biol. 16 (12), e1008543. 10.1371/journal.pcbi.1008543 33378330 PMC7802934

[B7] Choudhury A. Mohammad T. Anjum F. Shafie A. Singh I. K. Abdullaev B. (2022). Comparative analysis of web-based programs for single amino acid substitutions in proteins. PLoS One 17 (5), e0267084. 10.1371/journal.pone.0267084 35507592 PMC9067658

[B8] David A. Sternberg M. J. E. (2023). Protein structure-based evaluation of missense variants: resources, challenges and future directions. Curr. Opin. Struct. Biol. 80, 102600. 10.1016/j.sbi.2023.102600 37126977

[B32] Dordoni C. Zeni L. Toso D. Mazza C. Mescia F. Cortinovis R. (2024). Monoallelic pathogenic IFT140 variants are a common cause of autosomal dominant polycystic kidney diseasespectrum phenotype Clin. Kidney. J. 17 (2), sfae026. 38404363 10.1093/ckj/sfae026 PMC10894029

[B9] Fowler D. M. Rehm H. L. (2024). Will variants of uncertain significance still exist in 2030? Am. J. Hum. Genet. 111 (1), 5–10. 10.1016/j.ajhg.2023.11.005 38086381 PMC10806733

[B10] Geoffroy V. Stoetzel C. Scheidecker S. Schaefer E. Perrault I. Bär S. (2018). Whole-genome sequencing in patients with ciliopathies uncovers a novel recurrent tandem duplication in IFT140. Hum. Mutat. 39 (7), 983–992. 10.1002/humu.23539 29688594

[B11] Henrie A. Hemphill S. E. Ruiz-Schultz N. Cushman B. DiStefano M. T. Azzariti D. (2018). ClinVar Miner: demonstrating utility of a Web-based tool for viewing and filtering ClinVar data. Hum. Mutat. 39 (8), 1051–1060. 10.1002/humu.23555 29790234 PMC6043391

[B12] Hollingsworth S. A. Dror R. O. (2018). Molecular dynamics simulation for all. Neuron 99 (6), 1129–1143. 10.1016/j.neuron.2018.08.011 30236283 PMC6209097

[B13] Khalifa M. Albadawy M. (2024). Artificial intelligence for clinical prediction: exploring key domains and essential functions. Comput. Meth. Prog. Bio. 5, 100148. 10.1016/j.cmpbup.2024.100148

[B14] Nguengang S. Lambert D. M. Olry A. Rodwell C. Gueydan C. Lanneau V. (2020). Estimating cumulative point prevalence of rare diseases: analysis of the Orphanet database. Eur. J. Hum. Genet. 28 (2), 165–173. 10.1038/s41431-019-0508-0 31527858 PMC6974615

[B15] Oud M. M. Latour B. L. Bakey Z. Letteboer S. J. Lugtenberg D. Wu K. M. (2018). Cellular ciliary phenotyping indicates pathogenicity of novel variants in IFT140 and confirms a Mainzer-Saldino syndrome diagnosis. Cilia 7, 1. 10.1186/s13630-018-0055-2 30479745 PMC6247778

[B16] Pan Z. Theesfeld C. L. (2024). Deciphering missense coding variants with AlphaMissense. Kidney Int. 106 (2), 175–178. 10.1016/j.kint.2024.02.022 38647510

[B17] Patel S. H. Bakhsh S. Conboy E. Hajrasouliha A. R. (2023). Case of ift140-associated mainzer saldino syndrome. Ophthalmic Genet. 44 (2), 208–210. 10.1080/13816810.2022.2113545 36063079

[B18] Perrault I. Saunier S. Hanein S. Filhol E. Bizet A. A. Collins F. (2012). Mainzer-Saldino syndrome is a ciliopathy caused by IFT140 mutations. Am. J. Hum. Genet. 90 (5), 864–870. 10.1016/j.ajhg.2012.03.006 22503633 PMC3376548

[B19] Pires D. E. Ascher D. B. Blundell T. L. (2014). mCSM: predicting the effects of mutations in proteins using graph-based signatures. Bioinformatics 30 (3), 335–342. 10.1093/bioinformatics/btt691 24281696 PMC3904523

[B20] Quazi S. (2022). Artificial intelligence and machine learning in precision and genomic medicine. Med. Oncol. 39, 120. 10.1007/s12032-022-01711-1 35704152 PMC9198206

[B21] Richards S. Aziz N. Bale S. Bick D. Das S. Gastier-Foster J. (2015). Standards and guidelines for the interpretation of sequence variants: a joint consensus recommendation of the. American college of medical genetics and genomics and the association for molecular pathology. Genet Med. 17 (5), 405–424. 10.1038/gim.2015.30 25741868 PMC4544753

[B22] Riedhammer K. M. Nguyen T. T. Koşukcu C. Calzada-Wack J. Li Y. Assia Batzir N. (2024). Implication of transcription factor FOXD2 dysfunction in syndromic congenital anomalies of the kidney and urinary tract (CAKUT). Kidney Int. 105 (4), 844–864. 10.1016/j.kint.2023.11.032 38154558 PMC10957342

[B23] Rodrigues C. H. M. Pires D. E. V. Ascher D. B. (2021). DynaMut2: assessing changes in stability and flexibility upon single and multiple point missense mutations. Protein Sci. 30 (1), 60–69. 10.1002/pro.3942 32881105 PMC7737773

[B24] Sapozhnikov Y. Patel J. S. Ytreberg F. M. Miller C. R. (2023). Statistical modeling to quantify the uncertainty of FoldX-predicted protein folding and binding stability. BMC Bioinforma. 24 (1), 426. 10.1186/s12859-023-05537-0 PMC10642056 37953256

[B25] Saygılı S. Koşukcu C. Baştuğ T. Doğan Ö. A. Yılmaz E. K. Kalyoncu A. U. (2023). A novel homozygous missense variant in TBC1D31 in a consanguineous family with congenital anomalies of the kidney and urinary tract (CAKUT). Clin. Genet. 104 (6), 679–685. 10.1111/cge.14406 37468454

[B26] Schmidts M. Frank V. Eisenberger T. Turki S. Bizet A. Antony D. (2013). Combined NGS approaches identify mutations in the intraflagellar transport gene IFT140 in skeletal ciliopathies with early progressive kidney Disease. Hum. Mutat. 34 (5), 714–724. 10.1002/humu.22294 23418020 PMC4226634

[B27] Schymkowitz J. Borg J. Stricher F. Nys R. Rousseau F. Serrano L. (2005). The FoldX web server: an online force field. Nucleic Acids Res. 33 (Web Server issue), W382–W388. 10.1093/nar/gki387 15980494 PMC1160148

[B28] Soyaltın E. Kasap-Demir B. Alparslan C. Arslansoyu-Camlar S. Perihan K. O. Kırbıyık Ö. (2018). Can a hand radiograph indicate a special diagnosis in a child with chronic kidney disease? Answers. Pediatr. Nephrol. 33 (5), 801–803. 10.1007/s00467-017-3742-0 28741273

[B29] Şükür E. D. K. Timucin E. Baştuğ T. Ozaltin F. (2025). A novel NUP85 variant expanding the phenotypic spectrum of NUP85-associated steroid-resistant nephrotic syndrome. Clin. Genet. 10.1111/cge.14703 39949197

[B30] Walsh N. Cooper A. Dockery A. O'Byrne J. J. (2024). Variant reclassification and clinical implications. J. Med. Genet. 61 (3), 207–211. 10.1136/jmg-2023-109488 38296635

[B31] Yeh T. C. Niu D. M. Cheng H. C. Chen Y. R. Chen L. Z. Tsui S. P. (2022). Novel mutation of IFT140 in an infant with Mainzer-Saldino syndrome presenting with retinal dystrophy. Mol. Genet. Metab. Rep. 33, 100937. 10.1016/j.ymgmr.2022.100937 36393898 PMC9646644

