# Supplementary figures and images for: Systematic use of protein free energy changes for classifying variants of uncertain significance: the case of IFT140 in Mainzer-Saldino Syndrome
Source: Front Mol Biosci. 2025 Apr 23;12:1561380. doi: 10.3389/fmolb.2025.1561380 (PMC12055525; doi:10.3389/fmolb.2025.1561380)

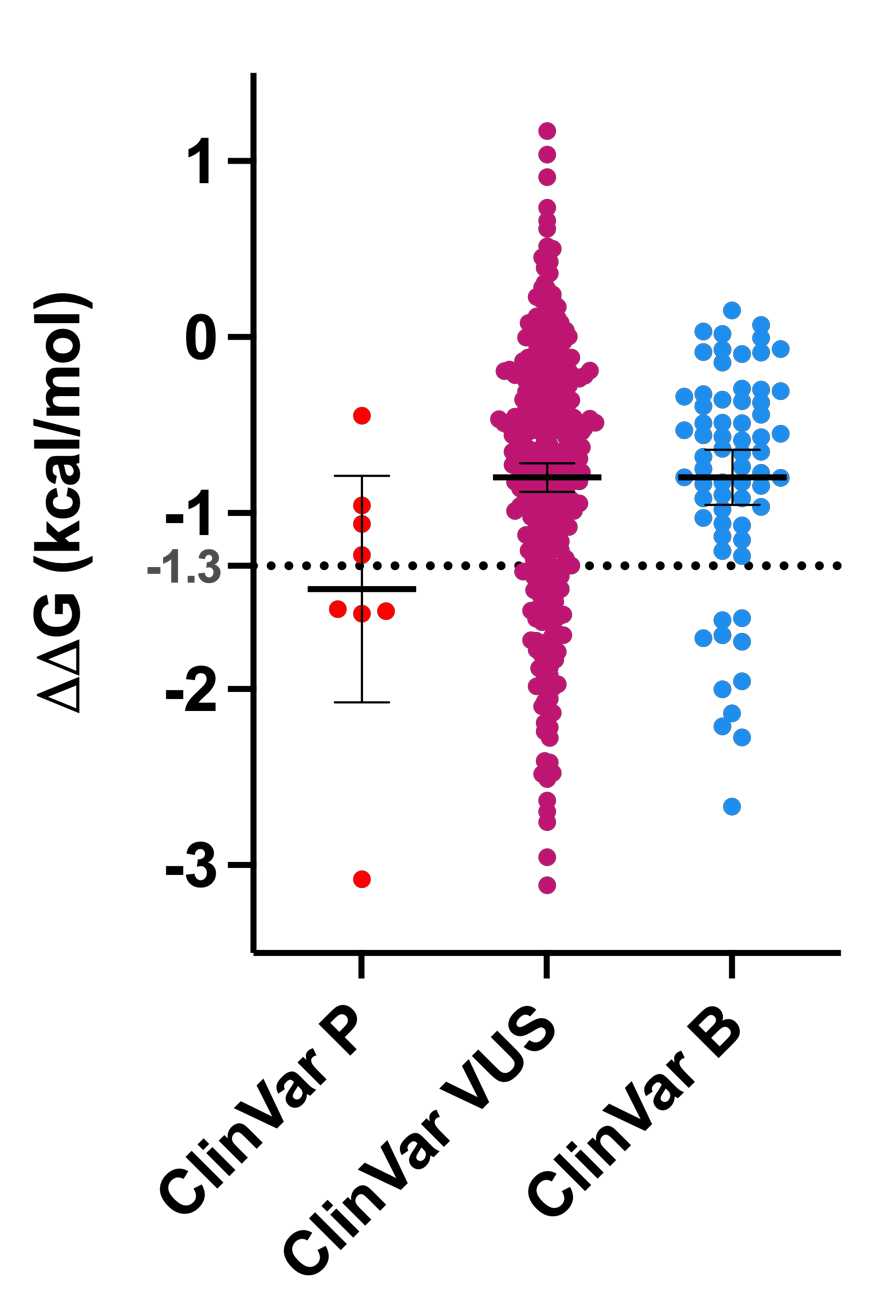

Supplement: Supplementary file 1 [file Image1.tiff]
